# Upregulation of microRNA-451 increases cisplatin sensitivity of non-small cell lung cancer cell line (A549)

**DOI:** 10.1186/1756-9966-30-20

**Published:** 2011-02-17

**Authors:** Hai-Bo Bian, Xuan Pan, Jin-Song Yang, Zhao-Xia Wang, Wei De

**Affiliations:** 1 Department of Oncology, The Second Affiliated Hospital of Nanjing Medical University, 121 Jiangjiayuan Road, Nanjing 210011, China; 2 Department of Oncology, Affiliated Nanjing First Hospital of Nanjing Medical University, 68 Changle Road, Nanjing 210006, China; 3 Department of Biochemistry and Molecular Biology, Nanjing Medical University, 140 Hanzhong Road, Nanjing 210029, China

## Abstract

**Background:**

Recently, miR-451 as a tumor suppressor has been reported in other studies. However, whether miR-451 can affect the sensitivity of non-small cell lung cancer (NSCLC) cells to cisplatin (DDP) remains unclear. The aim of this study is to evaluate the roles of miR-451 in the sensitivity of NSCLC cells to DDP.

**Methods:**

Quantitative RT-PCR assay was performed to detect the expression of miR-451 in 10 pairs of NSCLC and noncancerous tissue samples. pcDNA-GW/EmGFP-miR-451 was stably transfected into NSCLC cell line (A549). Then, the effects of miR-451 upregulation on growth, colony formation and apoptosis of A549 cells were investigated. Finally, the effects of miR-451 upregulation on in vitro and in vivo sensitivity of A549 cells of DDP were also determined.

**Results:**

The level of miR-451 expression in NSCLC tissues was significantly higher than that in corresponding noncancerous tissues. Ectopic overexpression of miR-451 could significantly inhibit growth and induce apoptosis of A549 cells. Moreover, ectopic overexpression of miR-451 could sensitize A549 cells to DDP possibly by increasing DDP-induced apoptosis which might be associated with the inactivation of Akt signaling pathway.

**Conclusions:**

This study demonstrated for the first time that combination of DDP application with miR-451 upregulation might be a potential strategy for the treatment of human NSCLC.

## Background

NSCLC accounts for the majority of lung cancer cases and chemotherapy has been the mainstay of treatments of lung cancers [1]. Up to date, DDP still remains the most widely used first-line chemotherapeutic agent for NSCLC treatment. However, continuous infusion or multiple administration of DDP often cause severe side effects, including myelosuppression, asthenia, and gastrointestinal disorders, as well as long-term cardiac, renal, and neurological consequences [2]. Therefore, improving the sensitivity to drug doses strategies is still a challenge for chemotherapy efficacy. Novel therapeutic modalities combining genetic and chemotherapeutic approaches will play important roles in the fight against cancer in future.

MicroRNAs (miRNAs) are small, endogenous non-coding RNAs that have been identified as post-transcriptional regulators of gene expression. MiRNAs exert their functions through imperfect base-pairing with the 3'-untranslated region (3'-UTR) of target mRNAs [3]. In human cancer, miRNAs can act as oncogenes or tumour suppressor genes during tumourigenesis. Evidence collected to date shows the involvement of microRNA and identifies this class of regulatory RNAs as diagnostic and prognostic cancer biomarkers, as well as additional therapeutic tools [4-6]. Meanwhile, the associations of dysregulation of miRNAs with chemoresistance of human cancers are attracting more and more attention [7]. Some researches have shown that dysregulation of miRNAs can contribute to the chemoresistance of cisplatin in human tumor cells [8,9]. Recently miR-451 has been reported to be induced during zebrafish, mouse, and human erythroid maturation as an key factor involved in regulates erythrocyte differentiation [10-12]. It was also reported that miR-451 might function as tumor suppressor and modulate MDR1/P-glycoprotein expression in human cancer cells [13]. Meanwhile, miR-451 has been reported to be involved in resistance of the MCF-7 breast cancer cells to chemotherapeutic drug doxorubicin [14]. However, to our best knowledge, there have been no reports about the association of miR-451 expression with the sensitivity of NSCLC cells to DDP.

In the present study, we identify miR-451 to be downregulated in human NSCLC and report for the first time that upregulation of miR-451 can enhance DDP chemosensitivity in NSCLC cell line (A549) by inducing apoptosis enhancement, which identifies miR-451 as a valid therapeutic target in strategies employing novel multimodality therapy for patients with NSCLC.

## Methods

### Patients and tissue samples

A total of 10 pairs of matched NSCLC and noncancerous tissue samples were surgically obtained from patients in Nanjing Chest Hospital, Jisnsu Province and diagnosed by an independent pathologist. None of the patients had received chemotherapy or radiotherapy before surgery. Samples were snap-frozen in liquid nitrogen and stored at -80°C until RNA extraction. Written informed consent was obtained from all patients before surgery.

### Cell culture

NSCLC cell line (A549) was cultured in Dulbecco's modified Eagle's medium (Invitrogen, Carlsbad, CA) supplemented with 10% fetal bovine serum, 100 U/mL penicillin, and 100 μg/mL streptomycin. All cell lines were cultured under the atmosphere of 5% CO_2 _with humidity at 37°C.

### Plasmid construction

The precursor sequence of miR-451 generated by annealing and primer extension with miR-451-precursor-F (5'-*TGCTGAAACCGTTACCATTACTGAGTTGTTTTGGCCACTGACTGA- CAACTCAGTTGGTAACGGTTT*-3') and miR-451-precursor-R (5'-*CCTGAAACCGTTACCAAC-TGAGTTGTCAGTCAGTGGCCAAAACAACTCAGTAATGGTAACGGTTTC*-3') was digested with BamHI and BglII and cloned into the BamHI-BglII fragment of the pcDNA-GW/EmGFP-miR vector (GenePharma, Shanghai, China). A construct including the non-specific miR-NC (99 bp) was used as a negative control. The constructed vectors were named pcDNA-GW/EmGFP-miR-451 and pcDNA-GW/EmGFP-miR-NC, respectively.

### Cell transfection

A549 cells were seeded into 6-well plates and transfected with the miR-415-expressing vector or the control vector expressing a non-specific miR-NC using Lipofectamine 2000 (Invitrogen), and were selected with spectinomycin (100 μg/ml) to generate two stable monoclonal cell lines (a miR-218 stable cell line, A549/miR-451, and a control stable cell line, A549/miR-NC).

### Quantitative real-time polymerase chain reaction (qRT-PCR) assay

Total RNA was extracted using TRIzol reagent (Invitrogen, CA, USA). Reverse-transcribed complementary DNA was synthesized with the Prime-Script RT reagent Kit (TaKaRa, Dalian, China). Realtime polymerase chain reaction (PCR) was performed with SYBR Premix Ex Taq (TaKaRa, Dalian, China). For miRNA detection, mature miR-451 was reverse-transcribed with specific RT primers (miR-451: 5'-*CTCAACTGGTGTCGTGGAGTCGGCAATTCAGTTGAGAAA-CTCAG*-3' and U6: 5'-*TGGTGTCGTGGAGTCG*-3') quantified with a TaqMan probe, and normalized by U6 small nuclear RNA using TaqMan miRNA assays (Applied Biosystems, CA).

### Stem-loop conventional RT-PCR assay

Total RNA was extracted using TRIzol reagent (Invitrogen, USA). Reverse-transcribed complementary DNA was synthesized with the Prime-Script RT reagent Kit (TaKaRa, Dalian, China). Conventional PCR was used to assay miRNA expression with the specific forward primers and the universal reverse primer complementary to the anchor primer. U6 was used as internal control (Invitrogen, USA). The PCR primers for mature miR-451 or U6 were designed as follows: miR-451 sense, 5'-*ACACTCCAGCTGGGAAACCGTTACCATTACT*-3' and reverse, 5'-*CTGGTGTCGTGGAGTCGGCAA*-3'. U6 sense, 5'- *CTCGCTTCGGCAGCACA*-3' and reverse, 5'-*AACGCTTCACGAATTTGCGT*-3'. Then, the RT-PCR products were electrophoresed through a 1.5% agarose gel with ethidium bromide. Signals were quantified by densitometric analysis using the Labworks Image Acquisition (UVP, Inc., Upland, CA).

### Western Blot assay

Thirty micrograms of protein extract were separated in a 15% SDS-polyacrylamide gel and electrophoretically transferred onto a PDVF membrane (Millipore, Netherlands). Membranes were blocked overnight with 5% non-fat dried milk and incubated for 2 h with antibodies to phospharylated Akt (pAkt-473), total Akt, Bcl-2 and Bax (Santa Cruz Biotechnology, Santa Cruz, CA) and GAPDH (Sigma, USA). After washing with TBST (10 mM Tris, pH 8.0, 150 mMNaCl, and 0.1% Tween 20), the membranes were incubated for 1 h with horseradish peroxidase-linked goat-anti-rabbit antibody. The membranes were washed again with TBST, and the proteins were visualized using ECL chemiluminescence and exposed to x-ray film.

### 3-(4,5-dimethylthazol-2-yl)-2,5-diphenyltetrazolium bromide (MTT) assay

The mock or stably transfected A549 cells were seeded into 96-well plates (6.0 × 10^3 ^cells/well) and allowed to attach overnight. After cellular adhesion, freshly prepared anticancer drugs (DDP) were added with various concentrations. After 72 h, cell viability was assessed using MTT assay. The absorbance at 490 nm (A490) of each well was read on a spectrophotometer. Three independent experiments were performed in quadruplicate.

### Colony formation assay

Approximately 500 mock A549 or stable transfect A549 cells (A549/miR-451 and A549/miR-NC) were placed in a fresh 6-well plate with or without DDP for another 12 h and maintained in RMPI 1640 containing 10% FBS for 2 weeks. Colonies were fixed with methanol and stained with 0.1% crystal violet in 20% methanol for 15 min.

### Flow cytometry analysis of apoptosis

Cells were treated with or without DDP for another 12 h and harvested and fixed with 2.5% glutaraldehyde for 30 minutes. After routine embedment and section, the cells were observed under electronic microscope. The apoptosis rates were determined using Annexin V-FITC and PI staining flow cytometry.

### Hoechst staining assay

Cells were cultured on 6-well tissue culture plates to confluence and treated with or without DDP for another 12 h. Then, Hoechst 33342 (Sigma, USA) was added to the culture medium of living cells; changes in nuclear morphology were detected by fluorescence microscopy using a filter for Hoechst 33342 (365 nm). The percentages of Hoechst-positive nuclei per optical field (at least 50 fields) were counted.

### Caspase-3 activity

The activity of Caspase-3 was measured using Caspase-3 Colorimetric Assay Kit (Nanjing Keygen Biotech. Co., Ltd) following the manufacturer's instruction. In brief, cells were seeded in the 6-wells and were cultured for 24 h. Then, the cells were administered with or without DDP for another 12 h and harvested, resuspended in 50 μL of lysis buffer and incubated on ice for 30 min, and cellular debris was pelleted. The lysates (50 μL) were transferred to 96-well plates. The lysates were added to 50 μL 2.0 × Reaction Buffer along with 5 μL Caspase-3 Substrate and incubated for 4 h at 37°C, 5% CO_2 _incubator. The activities were quantified spectrophotometrically at a wavelength of 405 nm.

### Terminal Transferase dUTP Nick End Labeling (TUNEL) Assay

Tissues were plated on polylysine-coated slides, fixed with 4% paraformaldehyde in 0.1 M phosphate-buffered saline (PBS) for 1 h at 25°C, rinsed with 0.1 M PBS, pH 7.4, and permeabilized with 1% Triton X-100 in 0.01 M citrate buffer (pH 6.0). DNA fragmentation was detected using TUNEL Apoptosis Detection Kit (Nanjing KeyGen, China), which specifically labeled 3'-hydroxyl termini of DNA strand breaks using fluorescein isothiocyanate (FITC)-conjugated dUTP. DNA was also labeled with FITC DNA-binding dye for 5 min. FITC labels were observed with a fluorescence microscope. The percentage of apoptotic cells was calculated as the number of apoptotic cells per number of total cells × 100%.

### Animal experiment

All experimental procedures involving animals were in accordance with the Guide for the Care and Use of Laboratory Animals and were performed according to the institutional ethical guidelines for animal experiment. Each aliquot of mock or stably transfected A549 cells were injected into the flanks of BALB/c nude mice (Nu/Nu, female, 4-6 weeks old) which were purchased from the Experimental Animal Centre of Nanjing Medical University and maintained under pathogen-free conditions (n = 8/group). One day after tumor cell implantation, mice were treated with CDDP (3.0 mg/kg body weight; i.p., thrice/week), Tumor volume was followed up for 4 weeks and measured once weekly. The tumor volume formed was calculated by the following formula: V = 0.4 × D × d^2 ^(V, volume; D, longitudinal diameter; d, latitudinal diameter). All mice were killed and s.c. tumors were resected and fixed in 10% PBS. TUNEL staining assay was performed on 5 μm sections of the excised tumors. The number of apoptotic cells in five random high-power fields was counted.

### Statistical analysis

All experimental data were shown as the mean ± SEM. Differences between samples were analyzed using the Student's *t *test. Statistical significance was accepted at *P *< 0.05.

## Results

### MiR-451 is significantly downregulated in human NSCLC tissues

**Figure 1:**
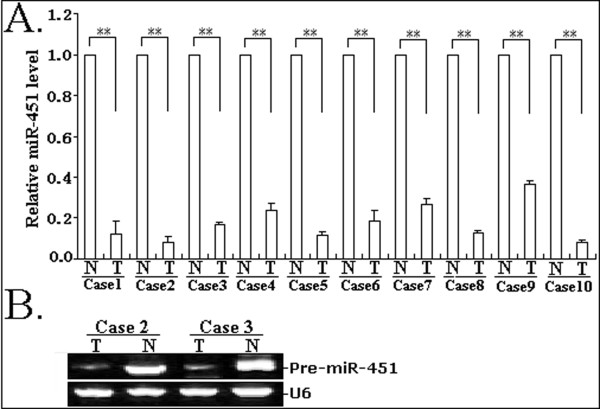
**Detection of miR-451 expression in tissue samples**. A. Quantitative RT-PCR analysis of miR-451 expression in 10 cases of NSCLC and corresponding noncancerous tissues. **P < 0.01. N: noncancerous tissues; T: tumor tissues. B. Conventional stem-loop RT-PCR analysis of miR-451 expression in NSCLC and corresponding noncancerous tissues. Gel images of electrophoresis. U6 was used as an internal control. All experiments were performed in triplicate.

In this study, a stem-loop qRT-PCR assay was performed to determine the expression of miR-451 in 10 pairs of matched NSCLC and noncancerous lung tissue samples. As shown in Figure 1A, the expression levels of miR-451in NSCLC tissues were less than approximately 36.4% of those in noncancerous lung tissues. In addition, conventional RT-PCR assay was also performed to analyze the expression of miR-451 in 2 pairs of matched NSCLC and noncancerous tissue samples. The gel electrophoresis of RT-PCR products confirmed the downregulation of miR-451 expression in NSCLC tissues (Figure 1B). Therefore, it was concluded that the downregulation of miR-451 might be involved in lung carcinogenesis.

### The expression of miR-451 could be significantlu upregulated in A549 cells by pcDNA-GW/miR-45

**Figure 2:**
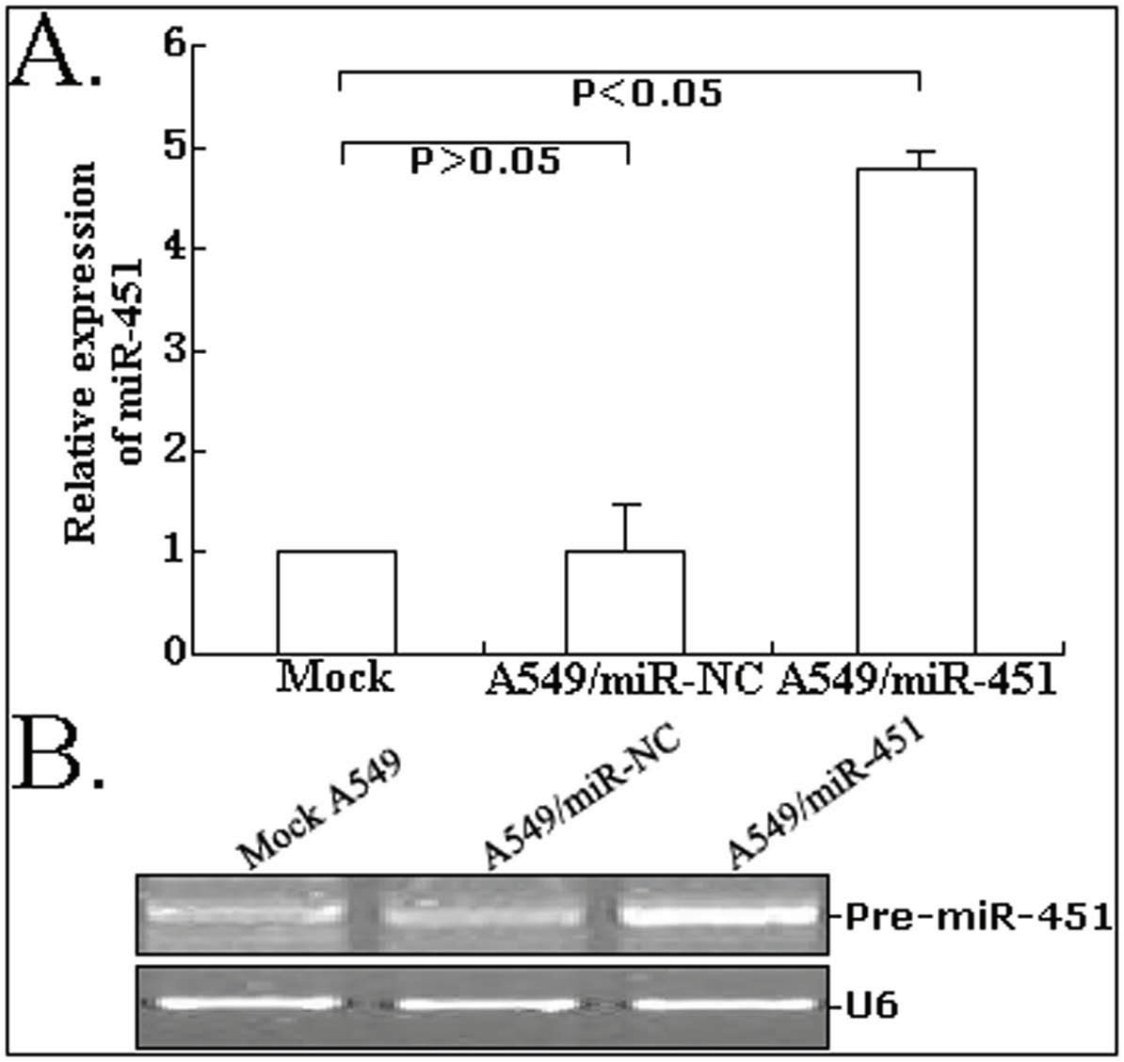
**Detection of miR-451 expression in mock or stably transfected A549 cells**. A. Quantitative RT-PCR analysis of miR-451 expression in A549, A549/miR-NC or A549/miR-451 cells. B. Conventional stem-loop RT-PCR analysis of miR-451 expression in A549, A549/miR-NC or A549/miR-451 cells. Gel images of electrophoresis. U6 was used as an internal control. All experiments were performed in triplicate.

To upregulate the expression of miR-451 in NSCLC cell line (A549), pcDNA-GW/miR-451 was transfected and stable transfectants (A549/miR-451 or A549/miR-NC) were successfully established. As shown in Figure 2A, qRT-PCR assay showed that the relative level of miR-451 expression in A549/miR-451 could be significantly upregulated by 3.8-fold compared with that in mock A549 or A549/miR-NC cells (*P *< 0.05). The gel electrophoresis of RT-PCR products confirmed the upregulation of miR-451 expression in A549/miR-451 cells (Figure 2B).

### Upregulation of miR-451 inhibits growth and enhances apoptosis of NSCLC cell line (A549)

**Figure 3:**
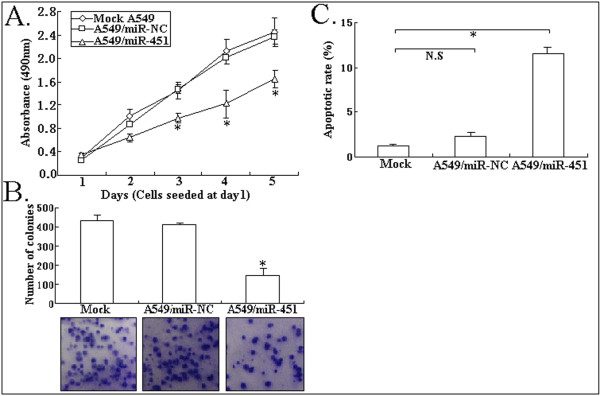
**Effect of miR-451 upregulation on growth and apoptosis of A549 cells**. A. MTT analysis of cell viability in mock A549, A549/miR-NC or A549/miR-451 cells. *P < 0.05. B. Detecting colony formation ability of mock A549, A549/miR-NC or A549/miR-451 cells, *P < 0.05. C. Flow cytomerty analysis of apoptosis in mock A549, A549/miR-NC or A549/miR-451, *P < 0.05; N.S, *P *> 0.05. All experiments were performed in triplicate.

To analyze the effect of miR-451 expression on phenotypes of NSCLC cell line, we performed MTT, colony formation and flow cytometric assays. As shown in Figure 3A, A549/miR-451 cell line had a significant increase in cell viability compared with mock A549 or A549/miR-NC cell line (*P *< 0.05). The number of colonies formed from A549/miR-451 cells was significantly lower than that formed from mock A549 or A549/miR-NC cells (*P *< 0.05; Figure 3B). Moreover, flow cytometric analysis showed that the apoptotic rate of A549/miR-451 cells (11.6 ± 1.5%) was significantly higher than that of mock A549 or A549/miR-NC cells (*P *< 0.05; Figure 3C). Thus, upregulation of miR-451 could induce growth inhibition and apoptosis enhancement in A549 cells.

### Upregulation of miR-451 expression inactivates the Akt signaling pathway of A549 cells

**Figure 4:**
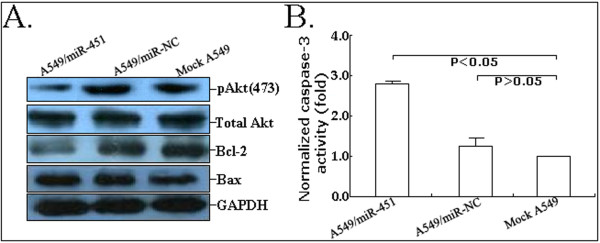
**Effect of miR-451 upregulation on the Akt signaling pathway**. A. Western Blot analysis of pAkt (473), total Akt, Bcl-2 and Bax protein expression in mock A549, A549/miR-NC or A549/miR-451 cells. GAPDH was used as an internal control. B. Analysis of relative caspase-3 activity in mock A549, A549/miR-NC or A549/miR-451 cells. All experiments were performed in triplicate.

It has been reported that activation of the Akt signaling pathway can regulate many biological phenomena of lung cancer cells, such as cell proliferation and survival, motility and migration. Thus, we analyzed the effects of miR-451 on the Akt signaling pathway in A549 cells (Figure 4A). Results showed that the upregulation of miR-451 could significantly downregulate the expression of pAkt protein but had no effects on the expression of total Akt protein. Additionally, the expression of Bcl-2 protein was downregulated and the expression of Bax protein was upregulated. The activity of caspase-3 in A549/miR-451 cells was also found to be significantly enhanced compared with that in mock A549 or A549/miR-NC cells (*P *< 0.05; Figure 4B). Therefore, it was concluded that the elevation of caspase-3 activity might be induced by the elevated ratio of Bax/Bcl-2. However, the exact mechanisms of miR-451 affecting the Akt signaling pathway need to be elucidated in future.

### Upregulation of miR-451 enhances in vitro sensitivity of A549 cells to DDP

**Figure 5:**
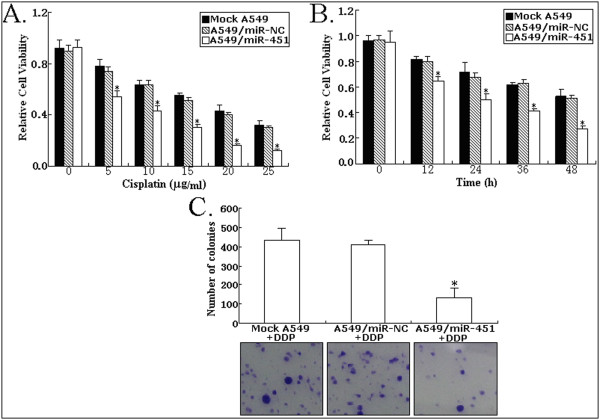
**Effect of miR-451 upregulation on the in vitro sensitivity of A549 cells to DDP**. A. Effects of various concentrations (0, 5, 10, 15, 20 and 25 μg/ml) of DDP on cells (mock A549, A549/miR-NC or A549/miR-451) for 12 h assessed by MTT assay. B. Effects of 5 μg/ml DDP on cells (mock A549, A549/miR-NC or A549/miR-451) for varied time length (0, 12, 24, 36 and 48 h) evaluated by MTT assays. C. Effects of 5 μg/ml DDP on colony formation of cells (mock A549, A549/miR-NC or A549/miR-451). All experiments were performed in triplicate, *P < 0.05.

Dysregulation of miRNA expression has been reported to be associated with chemoresistance of human cancers. However, whether miR-451 expression affects the sensitivity of NSCLC cells is not fully understood. To determine this, the mock or stably transfected A549 cells were treated with various concentrations (0, 5, 10, 15, 20 and 25 μg/ml) of DDP for 12 h or 5 μg/ml of DDP for 0, 12, 24, 26 and 48 h. The results from MTT assay indicated that upregulation of miR-451 led to a significant decrease in cell viability of A549 cells in response to DDP in a dose- or time -dependent manner compared with those of A549/miR-NC and mock A549 cells (Figure 5A and 5B). The cells were treated 5 μg/ml DDP for 12 h and the number of colonies was determined. As shown in Figure 5C, the number of colonies formed from A549/miR-451 cells treated with DDP was significantly lower than that formed from A549/miR-NC and mock A549 cells (P < 0.05). These data obviously showed that upresgulation of miR-451 might effectively enhance the sensitivity of A549 cells to DDP.

### Upregulation of miR-451 enhances DDP-induced apoptosis of A549 cells

**Figure 6:**
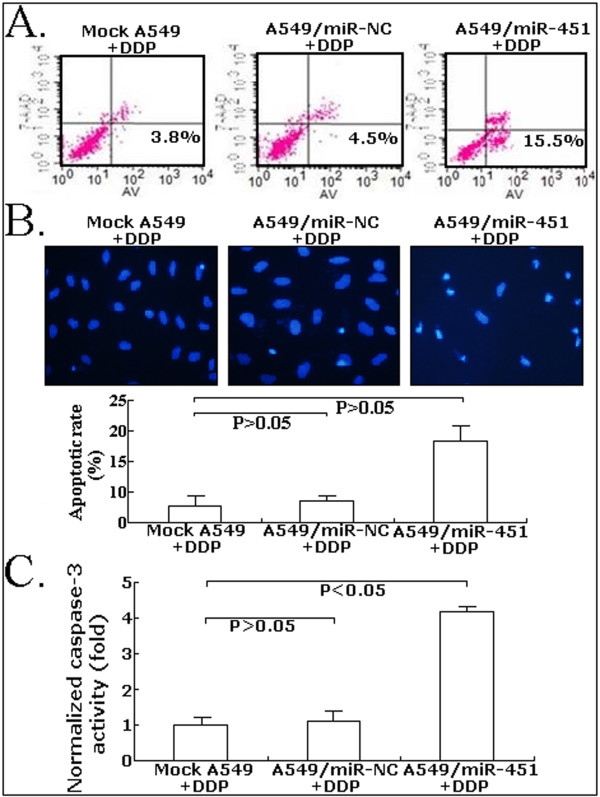
**Effect of combined miR-451 upregulation with DDP (5 μg/ml) on apoptosis of A549 cells**. A. Flow cytometry analysis of apoptosis in mock A549, A549/miR-NC or A549/miR-451 cells. B. Hoechst staining analysis of apoptosis in mock A549, A549/miR-NC or A549/miR-451 cells. C. Analysis of relative caspase-3 activity in mock A549, A549/miR-NC or A549/miR-451 cells. All experiments were performed in triplicate.

The precise underlying mechanisms by which upregulation of miR-451 enhances chemosensitivity of A549 cells to DDP were further investigated. Then, the apoptosis was detected by flow cytometric assay. As shown in Figure 6A, the apoptotic rare of A549/miR-451 treated with 5 μg/ml DDP was increased by approximately 11.7% in comparison with mock A549 cells treated with 5 μg/ml DDP (*P *< 0.05). However, the apoptotic rate of A549/miR-NC cells treated with DDP showed no significant difference compared with that of mock A549 cells treated with DDP (*P *> 0.05). Figure 6B showed the results of AnnexinV-FITC apoptosis detection assay, which confirmed the results of flow cytomeric assay. Finally, the activity of caspase-3 was also determined by colorimetric assay. As shown in Figure 6C, the caspase-3 activity in A549/miR-451 cells treated with DDP remarkably increased by approximately 308% compared that mock A549 or A549/miR-NC cells treated with DDP (*P *< 0.05). Therefore, upregulation of miR-451 might increase DDP chemosensitivity of A549 cells by enhancing DDP-induced apoptosis.

### Upregulation of miR-451 increases in vivo chemosensitivity of A549 cells to DDP

**Figure 7:**
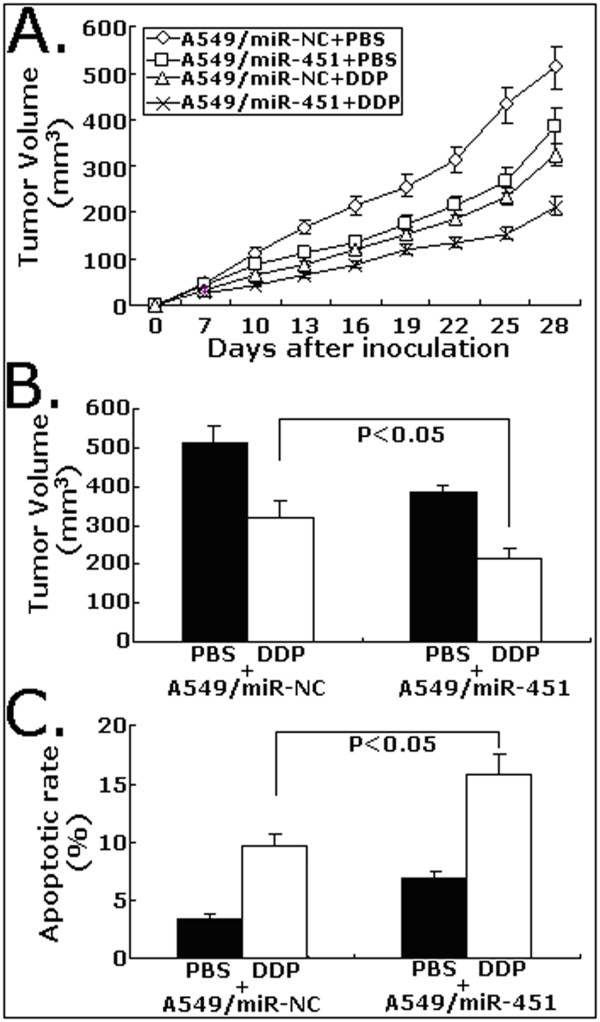
**Effect of miR-451 upregulation on the in vivo sensitivity of A549 cells to DDP**. A. Growth of tumors in the mice injected with A549/miR-451 or A549/miR-451 with or without DDP treatement. The inoculation was performed in eight mice. B. Average tumor volume at day 28 after the inoculation of A549/miR-NC or A549/miR-451 cells with or without DDP treatment (n = 8/group). C. TUNEL staining analysis of apoptosis in tumor tissues at day 28 after the inoculation of A549/miR-NC or A549/miR-451 cells with or without DDP treatment (n = 8/group).

To explore whether upregulation of miR-451 on chemosensitivity of A549 cells to DDP in vivo, s.c. tumors were developed in nude mice followed by treatment with DDP or PBS. As shown in Figure 7A, the tumors formed from A549/miR-451cells grew significantly slower than those from A549/miR-NC after the treatment with DDP. At 28 days after inoculation, the average tumor volume of A549/miR-451 cells (212 ± 36 mm^3^) was significantly lower than that of A549/miR-NC (323 ± 13 mm^3^) following DDP treatment (*P *< 0.05; Figure 7B). TUNEL assay showed that the apoptotic rate of tumors developed from A549/miR-451 cells (15.8 ± 2.2%) was significantly higher than that of tumors developed from A549/miR-NC cells (9.6 ± 1.5%) following DDP treatment (*P *< 0.05; Figure 7C). Like the results observed from in vitro experiments, upregulation of miR-451 could also increase in vivo chemosensitivity of A549 cells to DDP by inducing apoptosis enhancement.

## Discussion

MiRNAs are a growing class of small, noncoding RNAs (17-27 nucleotides) that regulate gene expression by targeting mRNAs for translational repression, degradation, or both. Increasing evidence suggests that deregulation of miRNAs has been frequently observed in tumor tissues. These miRNAs have regulatory roles in the pathogenesis of cancer in humans, through the suppression of genes involved in cell proliferation, differentiation, apoptosis, metastasis and resistance [15-18]. Recently, many studies have shown that miRNAs play an important role in malignant transformation. It is likely, therefore, that they can also modulate sensitivity and resistance to anticancer drugs in substantial ways. The mechanisms responsible for chemotherapy resistance by miRNAs have not been clearly identified. Current published data on the association of miRNAs with chemoresistance are limited. While altered expression of miRNAs in primary human NSCLCs has been used for tumor diagnosis and prognosis [19], the potential involvement of miRNAs in induction of drug resistance, particularly, in cisplatin resistance has not been explored.

Here, we showed that miR-451 is frequently downregulated in human NSCLC tissues compared with corresponding noncancerous lung tissues, which is consistent with the results of Gao'et al [20]. It was also reported that microRNA-451 could regulate macrophage migration inhibitory factor production and proliferation of gastrointestinal cancer cells [21]. Nan and his colleagues revealed that miR-451 impacts glioblastoma cell proliferation, invasion and apoptosis, perhaps via regulation of the PI_3_K/AKT signaling pathway [22]. Thus, miR-451 was proposed as a tumor-suppressor of human cancers. In other reports, Godlewski and his colleagues showed that miRNA-451 regulates LKB1/AMPK signaling and allows adaptation to metabolic stress in glioma cells, which represents a fundamental mechanism that contributes to cellular adaptation in response to altered energy availability [23]. At the same time, they also identified a potential feedback loop between LKB1 and miR-451, which allows a sustained and robust response to glucose deprivation [24]. P-glycoprotein, which is the MDR1 gene product, confers cancer cell resistance to a broad range of chemotherapeutics. Zhu, et al demonstrate for the first time the roles of miRNAs in the regulation of drug resistance mediated by MDR1/P-glycoprotein, and suggest the potential for targeting miR-27a and miR-451 as a therapeutic strategy for modulating MDR in cancer cells [13]. Olga and his colleagues reported that the enforced increase of miR-451 levels in the MCF-7/DOX cells down-regulates expression of mdr1 and increases sensitivity of the MCF-7-resistant cancer cells to DOX [14]. All these data provide a strong rationale for the development of miRNA-based therapeutic strategies aiming to overcome chemoresistance of tumor cells. However, whether the expression of miR-451 can affect the sensitivity of lung cancer cells to DDP is still unclear.

In the present study, we found that the upregulation of miR-451 could significantly inhibit growth and colony formation of NSCLC cell line (A549). Upregulation of miR-451 could also enhance caspase-3-dependent apoptosis of A549 cells by inactivating the Akt signalling pathway which induced the reverse of Bcl-2/Bax ratio. Furthermore, upregulation of miR-451 could significantly increase the in vitro and in vivo sensitivity of A549 cells to DDP. To the best of our knowledge, we provided the first insight into the roles and possible mechanisms of miR-451 upregulation in chemosensitivity of A549 cells to DDP. These data suggest that appropriate combination of DDP application with miR-451 regulation might be a potential approach to NSCLC therapy. For higher-dose DDP would produce potentially serious toxic effects such as nephro- and ototoxicity would be increased, combination of DDP application with miR-451 upregulation for the treatment of NSCLC would contribute to lower-dose DDP administration and result in a reduction of DDP toxic side-effects. Although inhibition of Akt signal pathway has been reported to be able to improve chemotherapeutic effect of human tumor cells, whether upregulation of miR-451 enhance DDP chemosensitivity of A549 cells by inactivating the Akt signal pathway needs to be further elucidated. Moreover, only A549 cell line has been used in this study, further researches should be conducted on other cell lines to testify our experimental data.

In conclusion, upregulation of miR-451 could increase the sensitivity of A549 cells to DDP both in vitro and in vivo, suggesting that appropriate combination of DDP application with miR-451 upregulation might be a potential strategy for the treatment of human NSCLC in future.

## Competing interests

The authors declare that they have no competing interests.

## Authors' contributions

HBB and XP contributed to clinical data, samples collection, MTT, apoptosis and caspase-3 activity detection analyses and manuscript writing. JSY contributed to animal experiment. ZXW and WD were responsible for the study design and manuscript writing. All authors read and approved the final manuscript.
